# The Correlation between Demographical and Lifestyle Factors and Traditional Chinese Medicine Constitution among Macau Elderly Individuals

**DOI:** 10.1155/2021/5595235

**Published:** 2021-04-17

**Authors:** Qian Bai, Yaochen Chuang, Yonghua Zhao, Yao Wang, Pu Ge, Youhua Xu, Ying Bian

**Affiliations:** ^1^State Key Laboratory of Quality Research in Chinese Medicine, Institute of Chinese Medical Sciences, University of Macau, Taipa, Macau 999078, China; ^2^Faculty of Chinese Medicine, Macau University of Science and Technology, Macao 999078, China; ^3^Kiang Wu Nursing College of Macau, Macao 999078, China

## Abstract

**Objectives:**

To measure the distributed characteristics of Traditional Chinese Medicine (TCM) constitutions, as well as related factors with biased TCM constitutions among the elderly individuals in Macau.

**Methods:**

The elderly individuals from elderly healthcare centers located in Macao Peninsula, Taipa, and Coloane were selected as research samples. The basic information questionnaire and the Constitution in Chinese Medicine Questionnaire (CCMQ) for elderly were employed. Descriptive analysis was applied to illustrate demographical characteristics and TCM constitution distribution. Exploratory factor analysis (EFA) was conducted to explore potential factors influencing biased constitutions, and weight of each variable for constitution was further calculated.

**Results:**

A total of 313 participants were recruited. Eighty-six (27.48%) elderly were identified as balanced constitution; others were biased constitutions accounting for 72.52%. Distribution differences related to gender and age were identified among participants with unbalanced constitutions. Four biased constitutions were further analyzed with EFA. For qi-stagnation and yang-deficiency constitutions, three similar factors were determined in the domains of education, sleeping habits, and lifestyle behaviors, successively. Although four factors were identified in phlegm-dampness constitution, the latter two belonged to lifestyle behaviors and the former two were education and sleeping habits. For yin-deficiency constitution, education, tobacco-alcohol consumption, sleeping habits, and exercise were four dimensions of potential influential factors. Tobacco consumption, sleep, and exercise duration weighted the most for qi-stagnation constitution; sleep duration, education level, and sugar-containing beverage intake for phlegm-dampness; length of education, alcohol consumption, and education level for yang-deficiency constitution; and weekly exercise hours, sleep duration, and education level for yin-deficiency constitution.

**Conclusion:**

The prevalence rate of biased constitutions was relatively high among elderly residents in Macau. Relations between demographical and lifestyle behavioral factors and biased constitutions were identified in this study. Controlling these influential factors might be beneficial for health management of Macau elderly individuals.

## 1. Introduction

According to data from World Health Organization, unpredictable health challenges have emerged, as the elderly population accelerates [1]. In China, by 2019, there were over 176 million people aged 65 years or above, making up 12.6% of the total population, which will reach up to 254 million accounting for 14.41% by 2030 as estimated [2]. Health issues induced by aging have imposed huge economic burden to the whole society. The elderly are susceptible to various chronic concerns, such as respiratory and cerebrovascular diseases, malignant tumors, hypertension, and diabetes [3]. The medical expenditure for elderly was approximately 1.43 trillion RMB in 2011, equivalent to 3.3 percentage of China GDP [4]. Therefore, it is of great necessity and significance to improve health status and decrease susceptibility to severe diseases in older people.

Constitution of traditional Chinese medicine (TCM) that originated from TCM theoretical system is proved to be efficient in population health management, especially for the elderly [5, 6]. TCM constitution proposed by Wang is one publicly recognized classification. Considering TCM theory and clinical practice, Wang classified constitutions into nine types, including balanced, yin-deficiency, yang-deficiency, qi-deficiency, blood stasis, phlegm-dampness, damp-heat, qi-stagnation, and inherited special constitutions [7]. TCM constitution is the comprehensive and stable manifestation of individual inherited, physical, physiological, and psychological condition. It determines the susceptibility, response, and progress of certain diseases; as a result, constitution theory can be applied to guide the prevention, treatment, and rehabilitation of diseases [8]. For example, people with phlegm-dampness constitution easily suffer from metabolic syndromes, such as obesity, hypertension, and hyperglycemia [9]; therefore, corresponding preventions for these diseases could be taken ahead. Balanced constitution is the ideal condition of human body with vigorous physique and strong resistance to endogenous and exogenous pathogens. Through harmonizing and regulating yin-yang, qi, blood, and other elements, eight biased constitutions could be improved towards balanced constitution and health-related quality of life is enhanced as a result [10].

Intrinsic endowment is the basis of TCM constitutions for human body, but it can be altered by external environments. Some studies have explored the correlation between acquired factors and constitutions. Wang's study stated that stress significantly affected the formation of some biased constitutions including yang- and yin-deficiency types among pregnant woman [11]. Additionally, lifestyle behaviors turn to be influential factors for certain constitutions; for instance, fried food is the predictor for yang- and yin-deficiency constitutions [12], while sleeping erratically and less physical exercise are risk factors for phlegm-dampness constitution [13]. Low and colleagues' research identified the link between constitutions and geographical aspects such percent greenery, total road intersection, percent road surface, and climate factors, and found place exerted important action on the development of body constitution [14]. Similarly, people in Guangzhou and Beijing showed significant differences in constitutions, which might be caused by lifestyle and living environments [15]. Those studies have provided scientific evidences for “constitution adjustable theory,” as well as the possibility of applying TCM constitution theory in health management through controlling influential risk factors.

As one Special Administrative Region of China, Macau is also bearing heavy health burden caused by aging. By the end of 2019, around 679 thousand residents are over 65 years old, accounting for 11.9% of the total population in Macau. The elderly people are tortured by cardiovascular diseases, osteoporosis, metabolic arthritis, and other illness, which lead to lower level of life satisfaction [16]. Although TCM constitution has been proved to be a valid measurement for health management, there are sparse comprehensive studies on TCM constitutions among Macau elderly residents, except for the fact that the constitution characteristics of reproductive older women in Macau were investigated by Zhang [17]. Therefore, we intended to analyze TCM constitution's distributed characteristics and explore influential factors related to biased constitutions among elderly people in Macau.

## 2. Methods

### 2.1. Participants

Sample size of this survey was calculated based on a single population proportion formula as follows [18]:

(1) $$\begin{array}{c} N=\frac{z^{2}p\left(1-p\right)}{e^{2}}. \end{array}$$

Sample size (*N*) was measured as 269 with 90% confidence level (*z* = 1.64), 0.50 maximum response distribution rate (*p*), and 0.05 margin of error (*e*). Considering the non-response rate, we set the sample size of 400 residents. Three elderly healthcare centers from Macao Peninsula, Taipa, and Coloane were selected as research sites. And questionnaires were sent to 400 elderly in these sites.

Inclusion criteria included (1) aged 65 years or above and (2) residents living in Macau for at least 25 years. Exclusion criteria included (1) complicated with severe systemic diseases, such as respiratory and heart failures, tumor, and mental disease and (2) being unable to complete verbal or written communication even with assistance.

### 2.2. Research Instruments

Ten investigators with TCM or nursing background interviewed samples and assisted them to complete survey. Basic information questionnaire and Constitution in Chinese Medicine Questionnaire (CCMQ) for elderly were used in this study. Basic information questionnaire contains demographical (i.e., gender, age, marital status, education background, and job condition), lifestyle behavior (i.e., sleep habits, exercise habits, tobacco and alcohol consumption, and dietary habits), and past medical history subdimensions. CCMQ for elderly is designed in accordance with the “TCM constitution classification standards” made by China Association of Chinese Medicine. It contains 33 items with 5-point Likert scale (from 1 (never happen) to 5 (always happen)). All items are organized into nine subscales, including balanced, qi-deficiency, yang-deficiency, yin-deficiency, phlegm-dampness, qi-stagnation, blood stasis, damp-heat, and inherited special constitutions. There are 5 items in balanced constitution with scores ranging from 5 to 25, and 4 items in each unbalanced constitution with scores ranging from 4 to 20. Some items are overlapping among subscales. If the score in the subscale of balanced constitution counted 17 or above, while score in the remaining eight biased constitutions was less than or equal to 10, then balanced constitution was identified. If the score in one unbalanced subscale was greater than 10, then the corresponding unbalanced constitution was determined; if the scores in more than one unbalanced subscale were greater than 10, then the larger one was chosen as the main constitution; and if the same score greater than 10 occurred in two or more unbalanced subscales, then a mixed constitution was identified.

### 2.3. Data Analysis

Six variables were identified as possible influential factors for biased constitutions in our study, including duration of stay in Macau, education level, length of education, time falling asleep, sleep duration, weekly exercise hours, tobacco consumption, alcohol consumption, and sugar-containing beverage intake. Sleep duration and weekly exercise hours are continuous variables, and the others are categorical rank variables.

Exploratory factor analysis (EFA) was employed to explore latent factors affecting eight biased constitutions. Principal components method was used for factor extraction, and varimax for factor rotation to obtain a more easily interpreted factor structure. Factor was identified when its eigenvalue was more than one. For each factor, variables with factor loading above 0.4 were retained. To ensure the reliability of EFA, we conducted the Kaiser-Meyer-Olkin (KMO) and Bartlett's test of sphericity. We set 0.5 as the minimum threshold of KMO value [19], and *p* value less than 0.05 in Bartlett's test indicated sufficient collinearity [20].

Weight of each variable was further calculated based on EFA results for each type of constitution. The equation was specified as follows [21]:

(2) $$\begin{array}{c} b_{i}=\frac{\sum_{j=1}^{m}\left.a_{ij}|/|\sqrt{\lambda_{i}}\right.*c_{j}}{\sum_{j=1}^{m}c_{j}}, \end{array}$$

where *b*_*i*_ represents the weight of each variable (*i*) for certain constitution; *a*_*ij*_ indicates the factor loading in the factor pattern of factors (*j*) and variables (*j*); *λ*_*i*_ means the eigenvalue of each factor; and *c*_*j*_ is the variance of separate factor.

The survey results were entered into Excel and data was cleaned in it. Then, all statistical procedures were performed in SAS 25.0 software.

## 3. Results

### 3.1. Basic Information of Participants

**Figure 1:**
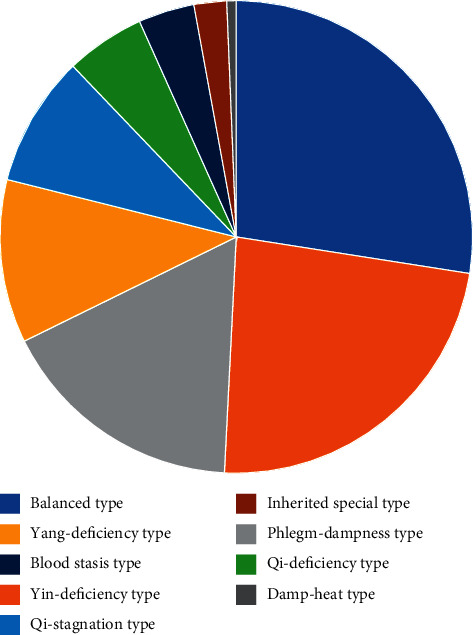
The distribution of TCM constitutions of included participants.

Of 400 interviewers, 56 were excluded because of incomplete information. Thirty-one participants were identified as mixed constitutions and the composition was diverse and irregular. Therefore, 31 participants who presented with mixed constitution were not analyzed in this research, and 313 participants were included for eventual analysis. Of 313 participants, 258 (82.43%) were female and 55 (17.57%) were male. The average age was (77.10 ± 8.22) years. Additionally, 11 (3.51%) participants were unmarried, 184 (58.79%) were married, and 118 (37.70%) were others. Only 9 (2.88%) participants graduated from specialty school or colleges, and others graduated from junior or primary school, even illiteracy. The most frequently occurring chronic diseases were hypertension (157, 50.16%), diabetes (69, 22.04%), and osteoporosis (66, 21.09%) in our samples (Table 1). In terms of constitution type, participants identified as balanced type (86, 27.48%) took up the largest proportion, followed by yin-deficiency type (73, 23.32%), phlegm-dampness type (53, 16.93%), yang-deficiency type (35, 11.18%), qi-stagnation type (28, 8.95%), qi-deficiency type (17, 5.43%), blood stasis type (12, 3.83%), inherited special type (7, 2.24%), and damp-heat type (2, 0.64%) (Figure 1).

### 3.2. The Distribution of Unbalanced TCM Constitutions by Gender and Age

**Figure 2:**
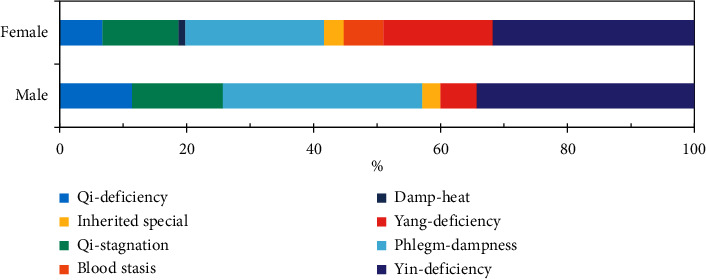
The distribution proportion of unbalanced constitutions by gender.

Among participants with unbalanced TCM constitutions, distribution difference related to gender was identified. There was larger proportion of yang-deficiency (33, 17.19%) in females compared with the proportion of 5.71% in males. Slightly higher proportion of blood stasis constitution was also found in females. On the contrary, the proportion of phlegm-dampness type in male outran that in female by nearly 10 percentage points, and similar difference also occurred in qi-deficiency constitution (Figure 2).

**Figure 3:**
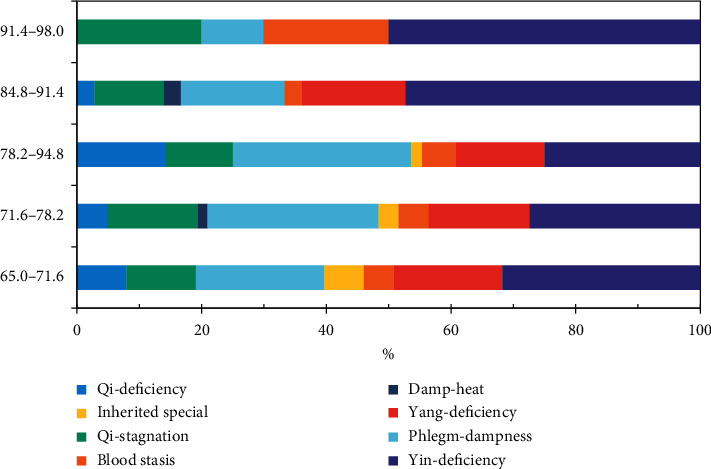
The distribution proportion of unbalanced constitutions by age.

To analyze the susceptibility towards certain constitution by ages, participants were classified into five age groups with equal interval as shown in Figure 3. It was noted that yin-deficiency constitution took up a relatively higher proportion in separate age group, and its proportion increased from 31.75% to 50.00% as the age group went up. Besides, the proportion of blood stasis constitution grew from 4.76% to 20.00% in the maximum age group.

### 3.3. Exploratory Factor Analysis

EFA was conducted to explore latent factors concerning eight biased constitutions, respectively. Results of qi-deficiency, blood stasis, inherited special, and damp-heat constitutions exceeded the threshold of KMO and Bartlett; therefore, only four biased constitutions were presented with EFA and weights calculation results.

#### 3.3.1. Qi-Stagnation Constitution

The KMO of qi-stagnation constitution EFA was 0.509 and Bartlett value was 70.743 (*p* < 0.05), which met the minimum requirements. Three factors were identified and explained 66.548% variance in total. Factor 1 included education level, length of education, and weekly exercise hours; Factor 2 included sleep duration, time falling asleep, and alcohol consumption; and Factor 3 included sugar-containing beverage intake, duration of stay in Macau, and tobacco consumption (Table 2).

#### 3.3.2. Phlegm-Dampness Constitution

The KMO measurement in EFA of phlegm-dampness constitution was 0.518, and Bartlett value reached 94.347 (*p* < 0.05). Four factors were retained with cumulative variance of 70.726%. Factor 1 contained education level and length of education; Factor 2 contained time falling asleep, sleep duration, and duration of stay in Macau; Factor 3 contained weekly exercise hours and alcohol consumption; and Factor 4 contained sugar-containing beverage intake and tobacco consumption (Table 3).

#### 3.3.3. Yang-Deficiency Constitution

All participants who manifested as yang-deficiency constitution had no habit of tobacco consumption; thus, the variable “tobacco consumption” was not included in its EFA. The KMO and Bartlett's value were 0.550 and 60.497 (*p* < 0.05), respectively. Eigenvalues of the first three factors were over one and the cumulative variance was 64.057%. Factor 1 retained education level, length of exercise, and weekly exercise hours; Factor 2 retained sleep duration, time falling asleep, and alcohol consumption; and Factor 3 retained sugar-containing beverage intake and duration of stay in Macau (Table 4).

#### 3.3.4. Yin-Deficiency Constitution

The KMO and Bartlett's value in EFA of yin-deficiency constitution were 0.533 and 118.215 (*p* < 0.05), respectively. Four factors were retained, and their cumulative variance was 69.078%. Factor 1 involved length of education and education level; Factor 2 involved tobacco and alcohol consumption; Factor 3 involved sleep duration, time falling asleep, and sugar-containing beverage intake; and Factor 4 involved duration of stay in Macau and weekly exercise hours (Table 5).

### 3.4. Weight of Each Variable for Unbalanced TCM Constitutions

Nine variables weighted a bit differently among four constitutions. Tobacco consumption (19.35%), sleep duration (17.70%), and weekly exercise hours (13.67%) exerted relatively obvious impact on qi-stagnation constitution; sleep duration (18.46%), education level (15.65%), and sugar-containing beverage intake (13.36%) on phlegm-dampness constitution; length of education (20.90%), alcohol consumption (19.07%), and education level (18.87%) on yang-deficiency constitution, while weekly exercise hours (15.82%), sleep duration (13.97%), and education level (13.80%) on yin-deficiency constitution (Table 6).

## 4. Discussion

Based on sampling survey, we explored the distributed characteristics of TCM constitution among the elderly in Macau. Findings demonstrated that 27.48% of participants were identified as balanced constitutions, and the prevalence rate of biased constitutions was 72.52%, while a previous nationwide population survey showed that biased constitutions accounted for 67.86% [22], which was slightly lower than our result. Physiological function of organs was attenuated by years; therefore, the elderly would be more susceptible to illness and have a tendency towards biased constitution [23]. Among eight biased constitutions, yin-deficiency, phlegm-dampness, and yang-deficiency constitutions were dominant in samples, accounting for 23.32%, 16.93%, and 11.18%, respectively. The most common diseases were hypertension, diabetes, and osteoporosis based on investigation. Prior studies have identified that different TCM constitution is predisposed to certain diseases. For instance, individuals with yin-deficiency and phlegm-dampness constitutions tend to suffer from hypertension [24, 25], while those with yang-deficiency constitution are more easily attacked by osteoporosis [26, 27]. Therefore, it makes contributions to alleviate disease burdens through identifying influential factors of biased constitutions and adjusting human body towards balanced constitution.

Distinctive distributions concerning gender and age were measured among participants with unbalanced constitutions. Females had larger possibilities towards yang-deficiency and blood stasis constitutions, while males tended to manifest with damp-heat and qi-deficiency constitutions in this research. This was similar to two national TCM constitution surveys in China [22, 28]. According to TCM classics, yin preponderates yang in female body and the physiological functions such as menstruation and parturition tend to cause the stagnation of blood; therefore, yang-deficiency and blood stasis constitutions are more common in female [29]. For males, they are more likely to have the habit of tobacco and alcohol consumption and preferential for greasy and salty diet, which might induce the accumulation of phlegm-dampness in body [22]. For age difference, yin-deficiency constitution was the dominant manifestation in every age group, and its proportion increased by nearly 20 percentage points from the minimum to the maximum age group. Besides, a higher proportion of blood-stasis constitution in older age groups was found in this study. Similar findings have already been reported [30]. As the growth of age, kidney-essence gradually weakens, and circulations of qi and blood become not smooth, which might cause various chronic illness; therefore, yang-deficiency and blood-stasis constitutions tend to appear [31].

TCM constitution is distinctive manifestations of inherited and acquired features of human body [32] and affected by demographical characteristics of people. Previous study found that phlegm-dampness constitution was common in well-educated groups, while types of yin-deficiency and yang-deficiency were common in less-educated groups [22]. EFA results in our study showed education factor was obviously influential for qi-stagnation, phlegm-dampness, yang-deficiency, and yin-deficiency constitutions. Although people with higher education have more access to healthcare management knowledge, they engage in well-paid, but complex and challenging work, enormous stress from which will contribute to the formation of biased constitutions [33, 34]. Additionally, EFA and weight calculation showed that “duration of stay in Macau” was relatively influential in phlegm-dampness constitution compared with other three types. Macau is located in south of China, featuring humid, muggy, and rainy climate, which leads to the development of phlegm-dampness constitution.

The relation between sleep habits and constitutions was also found in this study. In Ling Shu (Miraculous Pivot), a classic literature of TCM theory, regular and sufficient sleep balances yin-yang and nourishes essence, qi, and spirit; otherwise, biased constitutions are prone to be developed. EFA results demonstrated that sleep habit was a latent factor affecting qi-stagnation, phlegm-dampness, and yang-deficiency constitutions with the second highest variance contribution. Based on weight calculation, sleep duration plays a relatively important role in phlegm-dampness, qi-stagnation, and yin-deficiency constitutions, and time falling asleep in phlegm-dampness and yang-deficiency constitutions. Zhu's study indicated that sleeping habits, especially sleeping early and getting up late, and sleeping erratically obviously increased the tendency of developing into phlegm-dampness constitution [13]. Another study showed that sleeping duration was negatively related to yang-deficiency, yin-deficiency, phlegm-dampness, qi-stagnation, and qi-deficiency constitutions [35], which are in accordance with our results. From weight calculation, exercise duration impacted yin-deficiency constitution more obviously, which was consistent with prior studies [36, 37]. Based on TCM theory, exercise contributes to the fluent flow of qi, blood, and essence, which nourishes yin of body. Deficiency of yin leading to hyperactivity of fire would disturb the mind. Therefore, people with yin-deficiency constitution should get away from too vigorous exercise, and relaxed exercises such as Tai Chi, Ba Duan Jin, and Qi Gong are better choices for their health maintenance [38].

Lifestyle behaviors also affect human body constitutions. Tobacco and alcohol consumption were identified as the second factor influencing yin-deficiency constitution in EFA results. Tobacco consumption exerted obvious impact on qi-stagnation constitution, while alcohol consumption had a distinctive influence on yang-deficiency constitution according to weight calculation. Based on TCM theory, smoking consumes lung qi and yin-fluids and affects the fluency of qi movement, which are the pathogenic mechanisms of yin-deficiency and qi-stagnation constitutions [39]. People with yin- or yang-deficiency constitutions had better get rid of drinking alcohol, because it possibly results in effulgent fire to consume yin, and subsequently deficiency of yin deteriorates yang [40]. For phlegm-dampness constitution, sugar-containing beverage intake took a relatively high proportion weight. Appropriate diet has the functions of nourishing internal organs and strengthening human body. However, excessive sweet and greasy intake cause dysfunction of spleen and stomach in transportation and transformation; thus, phlegm and dampness are generated in the body [41]. Proper and regular eating and drinking habits are effective for boosting human body and prolonging life span.

There are some limitations in our research. Firstly, the uneven distribution between female and male participants might cause bias in conclusion. For example, males generally have more tendency towards smoking or drinking than females. Less male samples may underestimate the impact of tobacco and alcohol consumption on constitutions. Secondly, sample amount for EFA is relatively small. But a prior study also indicated that EFA with a small sample size was not per definition grossly wrong [42]; thus, our results are still reliable and credible to some extent. Thirdly, we only analyzed four types of biased constitution, and other four need to be further discussed. Finally, the existence of relation between demographical and lifestyle factors is identified in our study. But how these factors influence constitution either positively or negatively needs to be explored. Therefore, larger and more well-designed population surveys are necessary for seeking specific impact of demographical and lifestyle factors on TCM constitutions.

## 5. Conclusion

Based on population research, we found that the prevalence rate of biased constitutions was 72.52% and yin-deficiency, phlegm-dampness, and yang-deficiency were the main biased constitutions among the elderly in Macau. Females tended to have yang-deficiency and blood-stasis constitutions, while males tended to have damp-heat and qi-deficiency constitutions. Besides, yin-deficiency and blood stasis constitutions became more evident as age increased. Both education factor and sleeping habit were correlated with qi-stagnation, phlegm-dampness, yang-deficiency, and yin-deficiency constitutions. Undesirable lifestyle behaviors also exerted obvious impact on TCM constitutions, including tobacco consumption with yin-deficiency and qi-stagnation constitutions, alcohol consumption with yin- and yang-deficiency constitutions, and sugar-containing beverage intake with phlegm-dampness constitution. EFA and weight calculation provide empirical evidence for the relations between demographical and lifestyle factors with TCM constitution. Based on “constitution adjustable theory,” biased constitutions can be adjusted and improved through controlling these influential factors to achieve an age-friendly society.

## Figures and Tables

**Table 1:** Basic information of recruited participants.

Items	*n* (%)
Gender	
Male	55 (17.57)
Female	258 (82.43)
Age	77.10 ± 8.22
Marriage status	
Unmarried	11 (3.51)
Married	184 (58.79)
Others	118 (37.70)
Education level	
Illiteracy	77 (24.60)
Primary school and below	174 (55.59)
Junior school	53 (16.93)
Specialty school	5 (1.60)
College	4 (1.28)
Top three chronic diseases	
Hypertension	157 (50.16)
Diabetes	69 (22.04)
Osteoporosis	66 (21.09)
Total	313 (100.00)

**Table 2:** The EFA results for qi-stagnation constitution.

Variables	Factor 1	Factor 2	Factor 3

Duration of stay in Macau	−0.306	−0.056	**0.698**
Education level	**0.914**	−0.149	−0.163
Length of education	**0.897**	−0.063	0.023
Time falling asleep	0.387	−**0.772**	−0.075
Sleep duration	0.182	**0.878**	0.183
Weekly exercise hours	−**0.458**	−0.059	−0.437
Tobacco consumption	0.321	0.448	**0.602**
Alcohol consumption	−0.095	**0.610**	−0.119
Sugar-containing beverage intake	0.014	−0.010	**0.811**
Eigenvalues	2.238	1.973	1.778
Cumulative variance (%)	24.871	46.789	66.548

**Table 3:** The EFA results for phlegm-dampness constitution.

Variables	Factor 1	Factor 2	Factor 3	Factor 4
Duration of stay in Macau	−0.175	−**0.607**	0.332	0.130
Education level	**0.932**	0.089	0.040	0.008
Length of education	**0.903**	−0.046	0.037	−0.106
Time falling asleep	0.07	−**0.846**	0.022	0.048
Sleep duration	0.023	**0.661**	0.369	0.298
Weekly exercise hours	−0.086	0.136	−**0.882**	−0.026
Tobacco consumption	0.335	0.342	0.251	−**0.536**
Alcohol consumption	0.026	0.367	**0.554**	−0.462
Sugar-containing beverage intake	0.006	0.14	0.079	**0.824**
Eigenvalue	1.84	1.82	1.405	1.300
Accumulative contribution rate (%)	20.45	40.673	56.285	70.726

**Table 4:** The EFA results for yang-deficiency constitution.

Variables	Factor 1	Factor 2	Factor 3
Duration of stay in Macau	−0.013	−0.247	**0.555**
Education level	**0.911**	0.078	0.029
Length of education	**0.881**	−0.004	0.274
Time falling asleep	0.044	**0.737**	−0.151
Sleep duration	0.053	−**0.750**	0.325
Weekly exercise hours	**0.662**	0.111	−0.505
Alcohol consumption	0.220	**0.571**	0.365
Sugar-containing beverage intake	0.106	−0.003	**0.777**
Eigenvalue	2.108	1.511	1.505
Accumulative contribution rate (%)	26.354	45.243	64.057

**Table 5:** The EFA results for yin-deficiency constitution.

Variables	Factor 1	Factor 2	Factor 3	Factor 4
Duration of stay in Macau	−0.211	−0.172	−0.059	**0.785**
Education level	**0.880**	0.006	−0.001	−0.014
Length of education	**0.886**	0.036	−0.087	0.009
Time falling asleep	0.405	0.089	−**0.712**	0.022
Sleep duration	−0.048	0.234	**0.804**	0.023
Weekly exercise hours	−0.227	−0.247	−0.059	−**0.677**
Tobacco consumption	−0.064	**0.861**	0.063	0.091
Alcohol consumption	0.086	**0.840**	−0.004	−0.048
Sugar-containing beverage intake	0.391	−0.114	**0.596**	−0.014
Eigenvalue	1.987	1.616	1.527	1.087
Accumulative contribution rate (%)	22.074	40.029	56.995	69.078

**Table 6:** Weight of each variable for qi-stagnation, phlegm-dampness, and yang- and yin-deficiency constitutions (%).

Variables	Qi-stagnation	Phlegm-dampness	Yang-deficiency	Yin-deficiency
Duration staying in Macau	3.99	5.59	4.65	2.57
Education level	9.52	15.65	18.87	13.80
Education duration	13.00	11.79	20.90	13.40
Time falling asleep	6.12	10.49	10.18	1.94
Sleep duration	17.70	18.46	5.79	13.97
Weekly exercise hours	13.67	10.95	6.21	15.82
Tobacco consumption	19.35	6.54	—	13.18
Alcohol consumption	5.62	7.17	19.07	12.70
Sugar-containing beverage intake	11.02	13.36	14.32	12.63
Total	100.00	100.00	100.00	100.00

## Data Availability

The data supporting the findings of the study are available from the corresponding author upon request.

## Abbreviations

TCM: Traditional Chinese Medicine
CCMQ: Constitution in Chinese Medicine Questionnaire
EFA: Exploratory factor analysis
KMO: Kaiser-Meyer-Olkin.
